# Supplementary material for: Causes and consequences of sex-chromosome turnovers in Diptera
Source: Evol Lett. 2026 Mar 12;10(3):253–68. doi: 10.1093/evlett/qrag003 (PMC13231274; doi:10.1093/evlett/qrag003)
Supplement: qrag003_Supplemental_Files [file qrag003_supplemental_files.zip › Supplementary_Datasets.zip!/Supplementary_Datasets/Dataset_2_Graphical_representation_outgroups/Culicidae_Anopheles_gambiae_chromosomes.pdf]

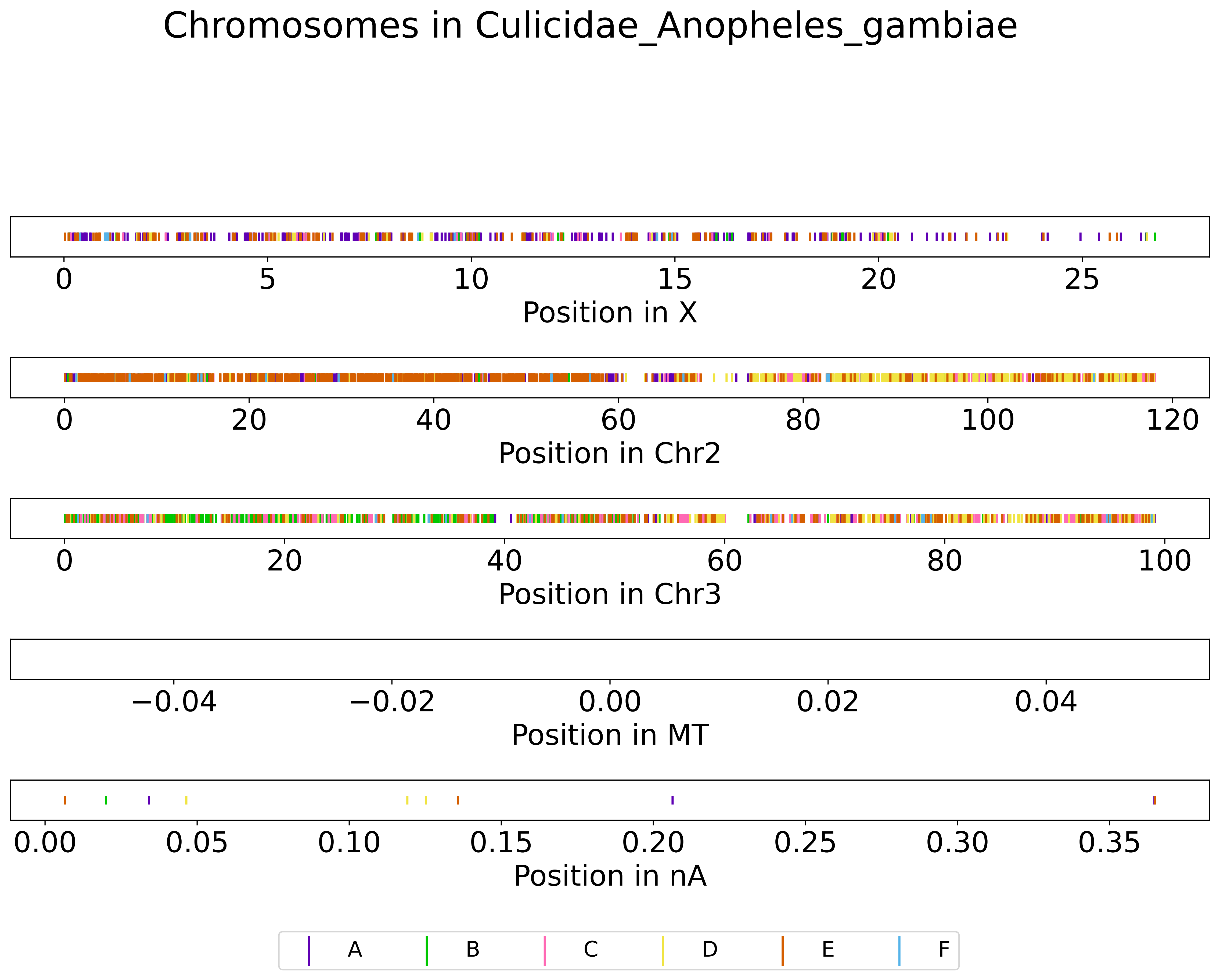
Chromosomes in Culicidae_Anopheles_gambiae
0
5
10
15
20
25
Position in X
0
20
40
60
80
100
120
Position in Chr2
0
20
40
60
80
100
Position in Chr3
−0.04
−0.02
0.00
0.02
0.04
Position in MT
0.00
0.05
0.10
0.15
0.20
0.25
0.30
0.35
Position in nA
A
B
C
D
E
F
